# Comparison of low and standard pressure gas injection at abdominal cavity on postoperative nausea and vomiting in laparoscopic cholecystectomy

**DOI:** 10.12669/pjms.305.5010

**Published:** 2014

**Authors:** Nozar Nasajiyan, Fatemeh Javaherfourosh, Ali ghomeishi, Reza Akhondzadeh, Faramarz Pazyar, Nader Hamoonpou

**Affiliations:** 1 Nozar Nasajiyan MD, Assistant Professor, Dept. of Anesthesiology, Ahvaz Jundishapur University of Medical Science, Pain Research Center, Ahvaz, Iran.; 2 Fatemeh Javaherfourosh MD, v Assistant Professor, Dept. of Anesthesiology, Ahvaz Jundishapur University of Medical Science, Pain Research Center, Ahvaz, Iran.; 3 Ali Ghomeishi MD, Assistant Professor, Dept. of Anesthesiology, Ahvaz Jundishapur University of Medical Science, Pain Research Center, Ahvaz, Iran.; 4 Reza Akhondzadeh , Assistant Professor, Dept. of Anesthesiology, Ahvaz Jundishapur University of Medical Science, Pain Research Center, Ahvaz, Iran.; 5 MD, Faramarz Pazyar MD, Assistant Professor, Dept. of Surgery, Ahvaz Jundishapur University of Medical Science, Ahvaz, Iran; 6 Nader Hamoonpou MD, Anesthesiologist, Dept. of Anesthesiology, Ahvaz Jundishapur University of Medical Science, Pain Research Center, Ahvaz, Iran

**Keywords:** Carbon dioxide, Cholecystectomy, Laparoscopy, Nausea, Pneumoperitoneum, Postoperative period, Pressure, Vomiting

## Abstract

***Background and Objective***: Postoperative nausea and vomiting (PONV) is the main concern for 40-70% of patients undergoing laparoscopic cholecystectomy. Our objective was to compare carbon dioxide gas at low pressure and standard pressure for the occurrence of PONV on patients undergoing laparoscopic cholecystectomy.

***Methods:*** This double- blind trial was conducted on 50 women patients aged between 18 to 60 years with acute cholecystectomy. The patients were divided into two groups: low pressure (LP) (received LP gas, 7-9 mmHg) and standard pressure (SP) (received SP gas, 14-15 mmHg). Nausea and vomiting in patients at hours 0-4, 4-8, 8-12, 12-24 after the surgery were recorded.

***Results:*** The frequency of PONV in the LP and SP groups did not demonstrate statistically significant different (*P *> 0.05). Nevertheless the frequency of shoulder pain after 4 hours at the LP group compared with SP group was significantly different (*P *< 0.023).

***Conclusions: ***The use of low pressure gas compared to standard pressure gas to create pneumoperitoneum could not reduce the PONV whereas the frequency of shoulder pain in LP group was reduced. Low pressure gas was associated with reduction of surgeon visibility and subsequently more prolonged surgery duration.

## INTRODUCTION

Laparoscopic cholecystectomy was introduced in 1992 as the gold standard intervention for gallstone operation.^1^ It can be associated with complications including dehydration, gastric aspiration and wound dehiscence.^2^

Almost 30 percent of all patients undergoing general anesthesia experience postoperative nausea and vomiting (PONV).^3^ PONV is the major distress of 40-70% of patients undergoing laparoscopic cholecystectomy.^4^ Although the precise mechanism of PONV is still unknown, we believe that high frequency of PONV in women undergoing laparoscopic operation may be due to pneumoperitoneum.^5^ Additionally, several cardiopulmonary changes occur during pneumoperitoneum, which is now introduced to allow laparoscopic cholecystectomy.^6^

Carbon dioxide (CO_2_) is noncombustible, quickly soluble in the blood, and relatively low-cost, so, it is the most common used gas for insufflation. Carbon dioxide is considered the major product of cellular metabolism and eliminated by a well-organized mechanism.^7^ Monitor of end tidal CO_2_ concentration is obligatory during the laparoscopy. It is suggested to employ the lowest intra-abdominal pressure allowing satisfactory exposure of the operative field, rather than using a routine pressure.^8^

The objective of this study was to find out the frequency of PONV in laparoscopic cholecystectomy at low pressure (8mm/Hg) vs. standard pressure (15mm/Hg) pneumoperitoneum.

## METHODS

***Study design and population: ***This double-blind trial was performed in a hospital affiliated to pain research center in Jundishapur University located in Ahvaz, Southwest Iran, from Dec. 2012 to Sep. 2013. A total of 50 women patients aged between 18 to 60 years old with acute cholecystitis were enrolled into the study. Exclusion criteria was defined as the patients who had cholangitis, history of motion sickness, malignancy, smoking, pregnancy and the patients who had received anti-emetic agents during the past 24 hours. Ethics Committee of Ahvaz Jundishapur University of Medical Sciences approved the study. A consent Form was received from the patients prior to the study.

***Intervention and measurement: ***Fifty qualified patients were randomly allocated for elective laparoscopic cholecystectomy to receive either standard pressure gas (14-15 mmHg) (n=25) or low pressure gas (7-9 mmHg) (n=25). Randomization was performed based on Color Cards (Blue & Red Cards).The patients belonged to class 1-2 in American Society of Anesthesiologist (ASA). All the laparoscopic cholecystectomies which performed by the same surgeon, standard monitoring and anesthetic management were standardized. Anesthesia was induced with: Midazolam 0.05 mg/kg, Fentanyl 2 mcg/kg, Thiopental 5 mg/kg and Cisatracurium 0.1 mg/kg. Mechanical ventilation was adjusted to maintain end-tidal CO_2_ tension 35-40 mmHg throughout the surgery. After induction of anesthesia, a nasogastric tube was passed into the stomach for suction and then removed. The patients were monitored for blood pressure and heart rate during the surgical procedure. At the end of the laparoscopic operations, surgeon was asked to state the quality of surgical field visualization. Also, when the patients were awake, questioner (who did not know the pressure gas) evaluated the nausea, vomiting and shoulder pain. The method used for pain control after operation was the infusion of Apotel 1 gr at recovery and repeat it after 6 hours and also, NSAIDs suppositories / PRN.

The patients were monitored for emetic symptoms within the first postoperative 24 hours for five times. Nausea and vomiting in patients were recorded at hours 0-4, 4-8, 8-12, 12-24, and after the surgery. Assessment of nausea intensity is difficult because the nausea is subjective, so in order to increase the accuracy of nausea assessment we used the visual analogue scale (VAS).^9 ^According to VAS, the number “zero” indicates that the patient does not feel any nausea and number “10” represents that the most severe level of nausea perception. [ 0-1 (no nausea), 1-4 (mild) , 4-7 (moderate) and 7-10 (severe)] .Vomiting was estimated based on frequency of emetic episode per 24 hours.[1-2 (mild) , 3-4 (moderate) and more from 5 (severe)]. The patients with persisted nausea for more than 20-30 min received Ondansetron 4 mg and were excluded the study**. **Also, shoulder pain in recovery room and 4 hours after the surgery were examined.

***Statistical considerations: ***The power of this study was considered 80%. Statistical analysis was performed using SPSS 19. The results were shown as mean ± standard deviation (SD) and 95% confidence intervals (CIs) of differences were calculated. We used Fisher exact and Chi Square tests to compare the variables between two groups. *P *< 0·05 was considered significant. 

## RESULTS

**Fig.1:**
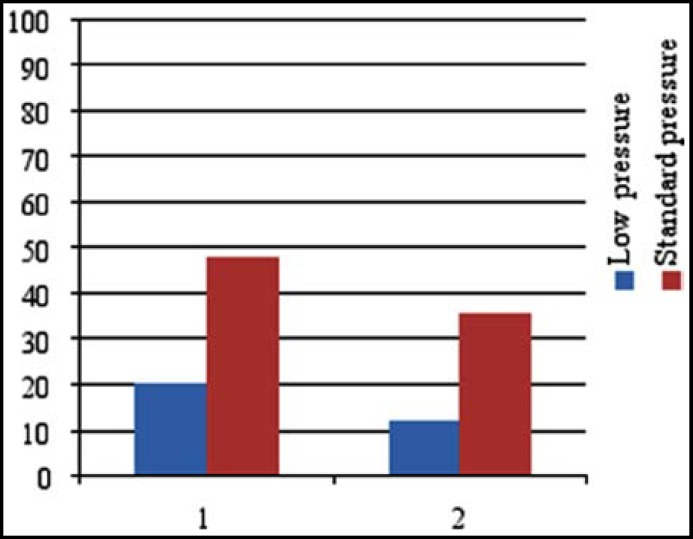
Comparison of low and standard pressure gas on shoulder pain at recovery room (1) and after 4 hours (2).

There was no significant difference between two groups based on demographic characteristics and surgical data, (P>0.05) (Table-I). The frequency of nausea and vomiting in patients at hours 0-4, 4-8, 8-12, 12-24, and after the surgery did not demonstrate statistically significant difference between LP and SP groups (P<0.05) (Tab2&3). In this study, the total dose of antiemetic medication (Ondansetron) used for moderate or severe nausea and vomiting were 30.65 and 34.80 mg respectively. Regarding the shoulder pain, the patients were questioned while leaving the recovery room and 4 hours after the operation. When leaving the recovery room, 5 and 12 patients in the LP and SP groups complained of shoulder pain, the difference was statistically significant (*P*> 0.023). Furthermore, 4 hours after the surgery, 3 and 9 patients in the LP and SP groups complained of shoulder pain, and the difference was significant (*P*> 0.005). (Fig.1)

## DISCUSSION

Open surgery has been substituted with laparoscopic cholecystectomy in the cholecystolithiasis treatment and it is now the gold standard in the treatment of benign gallstone disease.^10^^,^^11^ The risk of intraoperative injury during laparoscopic cholecystectomy is higher than open cholecystectomy.^12^ Laparoscopic surgery as a minimal invasive intervention requires pneumoperitoneum for adequate visualization and operative manipulation. Carbon dioxide is considered as the most frequent gas used for creating pneumoperitoneum because of high diffusibility and quick absorption and excretion.^13^ The use of CO_2_ in laparoscopy evokes local and systemic effects.^14^ Insufflations of CO_2_ into the peritoneum may change the acid-base balance, cardiovascular and pulmonary physiology, and in high risk patients they may elevate the postsurgical complications.^7^ Although the use of a proper gas pressure decreases the side effects, the reduction of gas pressure may interfere with the visualization of surgeons during the operation.^5^

**Table-I:** Demographic characteristics of patients and surgical data

	***Low pressure gas***	***Standard pressure gas***	***P***
Age (yr)	45.1±12.3	42.5±16.4	0.685
Weight (kg)	64.3±8.5	66.7±10.2	0.869
Duration of anesthesia (min)	145.25±20.5	138.30±18.8	0.532
Duration of surgery (min)	121.3±13.4	107.5±10.4	0.758
Pneumoperitoneum time (min)	101.8±9.2	93.4±11.4	0.256

**Table-II:** The frequency of nausea after surgery with low and standard pressure gas

***Time***	***Gas pressure***	***Mild nausea***	***Moderate nausea***	***Severe nausea***	***P***
0-4	Low Standard	4 4	4 7	1 3	0.500
8-4	Low Standard	3 4	1 3	0 1	0.160
8-12	Low Standard	3 5	2 3	1 2	0.269
12-24	Low Standard	0 0	3 3	0 2	0.351

**Table-III:** The frequency of vomiting after surgery with low and standard pressure gas

***Time***	***Gas pressure***	***Mild vomiting***	***Moderate vomiting***	***Severe vomiting***	***P***
0-4	Low Standard	2 2	1 2	0 1	0.702
8-4	Low Standard	3 2	0 2	0 0	0.356
8-12	Low Standard	0 1	0 1	0 0	0.490
12-24	Low Standard	0 0	0 0	0 0	0.875

PONV is frequent among the patients undergoing laparoscopic cholecystectomy. It can be very stressful to the patients, occasionally more than the operation itself.^2^ CO_2 _insufflations during laparoscopic surgery, causes peritoneal tension and irritation, playing main role in PONV.^15^

The first step in preventing of PONV after laparoscopic cholecystectomy is to decrease the patient risk factors, surgical intervention, anesthetic method, and postoperative cares.^4^ Different mechanisms have been mentioned for PONV after laparoscopic surgery. One of them is the dilation of brain vessels by CO_2_ increasing intra cranial pressure (ICP) and consequently the possibility of nausea and vomiting.^16^ Clinical and laboratory studies have revealed that ICP elevation is not directly relevant to the hemodynamic changes caused by the increased intra-abdominal pressure. The possible mechanism of increased ICP associated with insufflation may be impaired venous drainage of the lumbar venous plexus following increased intra-abdominal pressure.^17^ PONV is affected by many factors which related to patients, surgery and anesthesia. These variable items requires 5-Hydroxytryptamin (5.HT) secretion in a cascade of events concluding both the central nervous system and Gastro-intestinal tract.^18^ In addition, serotonin may play a role in pathogenesis of PONV. Various other mechanisms have been proposed for PONV after laparoscopic, including the effect of CO_2_ on the occurrence of ischemia in visceral tissues.^19^ PONV is decreased with the administration of high concentration oxygen,^20^ fluid therapy,^21^ and sympathomimetic agents.^22^

Likewise Propofol usage for anesthesia preservation has a positive effect on PONV reduction when N_2_O is administered as the unique anesthetic agent, it is recognized to cause of PONV. N_2_O can also be a reason of PONV because of change in middle ear pressure and bowel distension after diffusion in to closed cavities.^23^

In the present study, the use of low pressure gas instead of standard pressure gas could not significantly reduce the frequency of PONV after the surgery. Additionally, the duration of anesthesia, surgery and pneumoperitoneum were higher in the LP group; however, there were no significant differences. More prolonged duration of surgery was due to the reduction of visibility of the surgeon.

Kim *et al.* compared the low gas pressure with the standard gas pressure on PONV after laparoscopic gynecologic surgery. They reported that the use of low pressure CO_2 _was not effective in reducing the incidence and severity of PONV.^5^

In a study by Goll *et al. *administration of 80% oxygen during surgery was able to reduce the incidence of nausea and vomiting in gynecologic surgery.^24^ Researchers have illustrated that using CO_2_ to create pneumoperitoneum reduce 54% in the blood stream of the organs when using of intra- abdominal gas pressure (IAP) 15 mmHg instead of IAP 10 mmHg.^25^

A broad spectrum of adverse effects results from uncontrolled postsurgical pain.^26^ In our study shoulder pain after the surgery was assessed. When leaving the recovery room 20% and 48% of the patients experienced the shoulder pain in LP and SP groups respectively. Also, after 4 hours of surgery, 12% and 36% of the patients respectively complained the shoulder pain in LP and SP groups. Esmat et al. indicated that reduction of (CO2) pressure and the use of normal saline into the peritoneal membrane could reduce the frequency, percentage of shoulder pain after surgery in different hours after the operation from 35.2% to 18.4%.^27^

The present study had some limitations. We did not measure parameters involved in nausea and vomiting, including the amount of blood supply of the organs and tissue oxygenation. To obtain the most appropriate gas pressure, we suggest the assessment of gas pressure with less or higher rate than the standard range.

## CONCLUSION

In this study, the use of low pressure CO_2_ gas compared to standard pressure one to create pneumoperitoneum could not reduce nausea and vomiting after surgery. However, the frequency of shoulder pain in the low pressure gas group was reduced. Our findings demonstrated that during the operation, low pressure CO_2_ pneumoperitoneum was associated with problems including the reduction of surgeon visibility and subsequently more prolonged surgery duration. 
